# In silico analysis of single nucleotide polymorphism (rs34377097) of TBXA2R gene and pollen induced bronchial asthma susceptibility in West Bengal population, India

**DOI:** 10.3389/fimmu.2023.1089514

**Published:** 2023-03-01

**Authors:** Indranil Ganai, Ishita Saha, Priyajit Banerjee, Arghya Laha, Saheen Sultana, Nasima Sultana, Himani Biswas, Saibal Moitra, Sanjoy Podder

**Affiliations:** ^1^Ecology and Allergology Lab, Department of Zoology, The University of Burdwan, Burdwan, India; ^2^Department of Physiology, Medical College and Hospital, Kolkata, India; ^3^Fishery and Eco-toxicology Research Lab, Department of Zoology, The University of Burdwan, Burdwan, India; ^4^Post Graduate Department of Zoology, Krishnagar Government College, Krishnagar, India; ^5^Apollo Multispecialty Hospitals, Kolkata, India

**Keywords:** pollen sensitivity, asthma, thromboxane A2 receptor gene, SNP, FEV_1_/FVC ratio, homology modeling, West Bengal (India)

## Abstract

**Introduction:**

Prevalence of asthma is increasing steadily among general population in developing countries over past two decades. One of the causative agents of broncho-constriction in asthma is thromboxane A2 receptor (TBXA2R). However few studies of TBXA2R polymorphism were performed so far. The present study aimed to assess potential association of TBXA2R rs34377097 polymorphism causing missense substitution of Arginine to Leucine (R60L) among 482 patients diagnosed with pollen-induced asthma and 122 control participants from West Bengal, India. Also we performed in-silico analysis of mutated TBXA2R protein (R60L) using homology modeling.

**Methods:**

Clinical parameters like Forced expiratory volume in 1 second (FEV_1_), FEV_1_/Forced vital capacity (FVC) and Peak expiratory flow rate (PEFR) were assessed using spirometry. Patients’ sensitivity was measured by skin prick test (SPT) against 16 pollen allergens. Polymerase chain reaction-based Restriction fragment length polymorphism was done for genotyping. Structural model of wild type and homology model of polymorphic TBXA2R was generated using *AlphaFold2* and MODELLER respectively. Electrostatic surface potential was calculated using APBS plugin in PyMol.

**Results:**

Genotype frequencies differed significantly between the study groups (P=0.03). There was no significant deviation from Hardy-Weinberg equilibrium in control population (χ2=1.56). Asthmatic patients have significantly higher frequency of rs34377097TT genotype than control subjects (P=0.03). SPT of patients showed maximum sensitivity in *A. indica* (87.68%) followed by *C. nusifera* (83.29%) and *C. pulcherima* (74.94%). Significant difference existed for pollen sensitivity in adolescent and young adult (P=0.01) and between young and old adult (P=0.0003). Significant negative correlation was found between FEV1/FVC ratio and intensity of SPT reactions (P<0.0001). Significant association of FEV_1_, FEV_1_/FVC and PEFR was observed with pollen-induced asthma. Furthermore, risk allele T was found to be clinically correlated with lower FEV_1_/FVC ratio (P=0.015) in patients. Our data showed R60L polymorphism, which was conserved across mammals, significantly reduced positive electrostatic charge of polymorphic protein in cytoplasmic domain thus altered downstream pathway and induced asthma response.

**Discussion:**

The present in-silico study is the first one to report association of TBXA2R rs34377097 polymorphism in an Indian population. It may be used as prognostic marker of clinical response to asthma in West Bengal and possible target of therapeutics in future.

## Introduction

Asthma is correctly termed the 21st century epidemic and modern age disease and represents a substantial public health burden in many countries including India (1–3). The prevalence of asthma has been dramatically increasing in both developed and under developed countries across the world. According to Zvezdin 2015 (4), about 300 million people of all ages suffered from asthma. The disease severity is unevenly distributed, being mild in most cases, while severe asthma forms are registered in the minority of the patients (around 15%). It is assessed that another 100 million people will be affected by the disease until 2025 (5, 6). This lower airway disorder is not a new discovery, as it had been recognized over two hundred years ago. It is therefore amazing that it took centuries to realize the importance of disease diagnosis and adequate treatment. Estimate suggests that India alone reported 6% of children and 2% of adults suffering from asthma (3, 7). According to Global Burden of Disease (GBD, 1990–2019) study, India has 34.3 million asthmatics which accounts for 13.09% of the global burden.Evidence suggests that in Indian population, asthma accounted for 27.9% of disability-adjusted life years (DALYs) (8). One of the most significant causes of asthma was found to be natural exposure to pollens (9) that play a major role in its pathogenesis (10). An estimate suggested that thepollen induced asthma prevalence in India ranges between 10-40% of the total asthmatic population (11–14). Early detection of genetically predisposed individuals to asthma is important for better management of the disease. Bronchial asthma is characterized by several inflammatory mediators including histamine, bradykinin and arachidonic acid metabolites such as prostaglandins, thromboxane A2 (TBXA2), andcysteinyl leukotrienes (LTC4, LTD4, LTE4); responsible for the clinical and pathological events (15, 16). Over the last few decades, there has been a steady growth in interest in exploring the important roles of TBXA2 in the pathogenesis of asthma. TBXA2 binds to its receptor TBXA2R which is primarily expressed in tissues targeted by TBXA2, including bronchial and vascular smoothmuscle and platelets (17). TBXA2R stimulate broncho-constriction inhuman small airways (18) in response to pollen-induced mast cell activation in atopic asthma patients (19). It is a G protein-coupled receptor (GPCR) encoded by the TBXA2R gene located in Chromosome 19 at position p13.3 (20). Very few studies were recorded regarding the genetic association of TBXA2R with bronchial asthma so far.

So, in the present study, attempts have been made to investigate the association of TBXA2R rs34377097 polymorphism with pollen induced bronchial asthma among population of West Bengal, India. rs34377097 is the only non-synonymous polymorphism within TBXA2R gene reported till date (21). In this study, computational algorithms were performed on TBXA2R missense polymorphism (R60L) to examine the changes in protein structure and function. The change of amino acid from Arginine (R) to Leucine (L) may affect the protein conformation, leading to change in downstream signaling pathway. Therefore, the present study performed in-silico analyses of the mutated TBXA2R protein using homology modeling to explore and confirm the above outcome. Our study also attempted to determine the signaling molecules of TBXA2R pathway which are responsible for elevated asthmatic response in individuals carrying the risk allele.

## Materials and methods

### Ethics approval and consent to participate

The Clinical Research Ethics Committee of Allergy and Asthma Research Center, West Bengal, India approved the present study protocols vide CREC-AARC Ref: 62/21. Written informed consent was taken from each patient and control subjects of this study and preserved for future reference.

### Participants

A total of 632 individuals (both male and female) were selected for this case-control study during March to December, 2021. Pregnant and lactating women (n=5), patients suffering from illness other than asthma (n=11) and who did not provide the written informed consent (n=12) were excluded from this study. Finally, 482 patients and 122 control subjects were included in this present study. Details regarding epidemiology viz. age, gender, habitat, age of onset of symptoms, life-style, family history (Paternal or maternal), clinical features, aggravating factors and non-specific stimuli such as cold, exercise and other irritant factors, etc. were recorded in a self-prepared questionnaire. 482 participants diagnosed of having aero-allergen induced bronchial asthma and reported to be suffering from different asthmatic manifestations like airway hyper responsiveness, recurring episodes of airway obstruction, wheezing, dyspnoea and cough either alone or in different combination were considered as cases. Both patients and controls were classified according to their age like children (age 5–12 years), adolescents (age>12–19 years), young adults (age >19–40 years) and old adults (>40-70 years). Peripheral blood samples were collected from all the participants and centrifuged for separation of serum and kept in −20°C refrigerator for further analysis.

### Spirometry

The recruited subjects were screened for the presence of asthma by spirometry test following Global Initiative for Asthma (GINA) guidelines 2017 (22). The following parameters were measured: Forced expiratory volume at time interval of 1.0 second (FEV_1_), Forced vital capacity (FVC), FEV_1_/FVC ratio and Peak expiratory flow rate (PEFR). 20% or more reduction of FEV_1_ and PEFR value than predicted is considered as asthmatic (23). FEV_1_/FVC ratio less than 0.75-0.80 is considered as asthmatic (22).

### Skin prick test

Asthmatic patients those were positive in Spirometry test were undergone skin prick test (SPT) using 16 common aero allergen extracts (Credisol, Mumbai, India) viz. *Azadirachta indica*, *Cocos nucifera*, *Caesalpinia pulcherrima*, *Brassica nigra*, *Pinus sylvestris*, *Carica papaya*, *Zea mays*, *Triticum aestivum*, *Lantana camara*, *Poa pratensis*, *Chenopodium album*, *Peltophorum pterocarpum*, *Areca catechu*, *Parthenium hysterophorus*, *Lolium perenne* and *Plantago lanceolata*. Histamine phosphate and normal saline were used as positive and negative controls respectively. Wheel diameter ≥3mm was considered as positive response to that particular allergen.

### DNA extraction and genotyping

**Figure 1:**
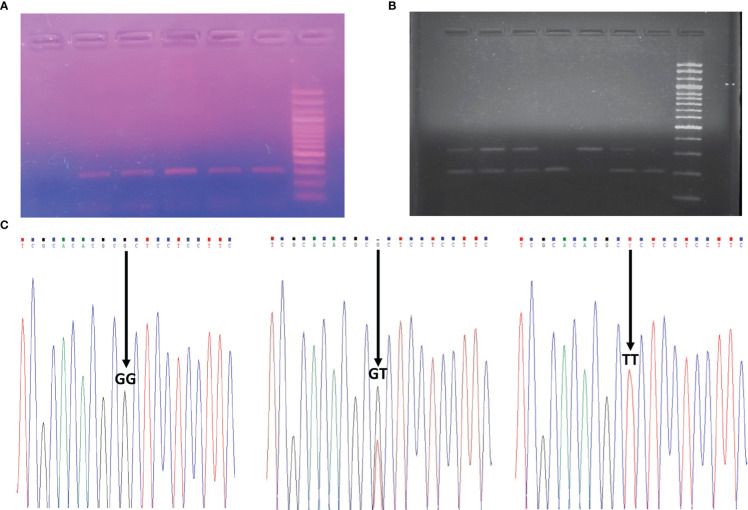
TBXA2R rs34377097 polymorphism. **(A)** PCR Product (311 bp). The last lane shows the 100 bp ladder. **(B)** RFLP fragments. GG genotype in Lane 4 and 7; GT genotype in Lane 1, 2, 3 and 6; TT genotype in Lane 5 **(C)** Representative chromatograms of GG, GT and TT genotype. The position of the genotypes is shown with an arrow.

Extraction of genomic DNA was performed from blood samples of 482 patients and 122 control subjects using QIAamp DNA blood mini kit (Qiagen, Hilden, Germany). TBXA2R rs34377097 polymorphism was identified using polymerase chain reaction (PCR) followed by restriction fragment length polymorphism (RFLP) technique (**Figure 1**). The details of both the primers, PCR conditions, PCR product, RFLP fragments are given in **Table 1**. Each reaction mixture (20µl) contains 50ng of genomic DNA, 200nM of each primer (Sigma), 200mM of each deoxyribonucleotide triphosphate (dNTP) (ThermoFisher Scientific, USA) and 2.5 units of Taq polymerase (Applied Biological Materials, Canada).

**Table 1:** PCR Conditions and restriction fragments of TBXA2R rs34377097 polymorphism.

SNP	Functional position	Primer sequence and PCR product size	PCR Condition	Restriction fragments and genotypes
TBXA2R rs34377097	Exon-2 G179T Arg to Leu Substitution (R60L)	Forward: 5’ TGGTGACTGATCCCTCAGG 3’ Reverse: 5’ TCGAAGAGCGCGGCGTGCT 3’ Product size- 311 bp	95 °C for 5 min, followed by 30 cycles at 95 °C for 30 s, 62°C for 30 s, 72 °C for 1 min, and a final extension at 72 °C for 10 min.	Enzyme: HhaI GG: 209, 102 bp TT: 311 bp GT: 311, 209, 102 bp

### DNA sequencing

Representative PCR products for each of the three polymorphic genotypes of the selected polymorphism were undergone Sanger sequencing to validate the RFLP results (**Figure 1**).

### *In silico* analysis

Structural model of wild type TBXA2R was generated using *AlphaFold2*. Homology model of TBXA2R R60L was generated using MODELLER (24, 25). Figures were designed using PyMol (26). The electrostatic surface potential was evaluated using APBS plugin in PyMol.T-Coffee server was used for multiple sequence alignment and data were prepared using ESPript (27).

### Statistical analysis

Continuous data are described by mean ± standard error (SE) and categorical data by number with percentages. Patients and controls were assessed by independent t test for continuous variables or Pearson chi-square test for categorical variables. Genotypic and allele distributionin patient and control group was evaluated using contingency chi-square or Fisher’s exact test, where applicable. Risk analysis was performed by odds ratio (OR) under additive, recessive and dominant models. Hardy-Weinberg equilibrium (HWE) was checked for the genotypes by goodness-of-fit chi-square test with one degree of freedom (df). SPT sensitivity for pollens among different age groups was analyzed using One way ANOVA followed by Tukey’s Multiple Comparison test. The association between FEV_1_/FVC ratio and atopy was analyzed using linear regression. FEV1/FVC ratio of both patient and control individuals residing in different habitats was analyzed using one way ANOVA test. The association of the TBXA2R polymorphic allele with FEV_1_/FVC ratio was analyzed using Independent t test. The association of the TBXA2R polymorphism with atopy was analyzed using one way ANOVA test. All statistical calculations were performed using GraphPad Prism ver. 7 (San Diego, CA). Significance level was taken as P<0.05.

## Results

### Characteristics of the subjects

The present study comprises of 482 asthmatic patients and 122 control individuals. Demographic and clinical characteristics are described in **Table 2**. Majority of the study population including patients and controls reside in urban area compared to sub-urban and rural ones. In case of FEV_1_, FEV_1_/FVC ratio and PEFR, a significant deviation was observed from the predicted value both in male and female patients in all age groups as compared to control (**Table 2**).

**Table 2:** Demographic and clinical characteristics of patients and control subjects.

Sl. No	Parameters	Category	Patients (n=482)			Control (n=122)	
1.	Age (Years)	Children(5-12 years) (Median 9 years)	128 (26.56%)			18 (14.75%)	
		Adolescents (13-19 years) (Median 16 years)	79 (16.39%)			30 (24.59%)	
		Young adults (20-40 years) (Median- 29 years)	184 (38.17 %)			46 (37.70%)	
		Old adults (40-70 years) (Median- 56.50 years)	91 (18.88%)			28 (22.95%)	
2.	Sex	Male	246 (51.04%)			56 (45.90%)	
		Female	236 (48.96%)			67 (54.92%)	
3.	Family history	Paternal (P)	97 (20.12%)			Nil	
		Maternal (M)	131 (27.18%)			Nil	
		P+M	44 (9.13%)			Nil	
		Absent	207 (42.95%)			122 (100%)	
4.	Weight (kg)		42.48±0.72 (41.06-43.90)			44.35±1.42(41.54-47.16)	
5.	Height(cm)		141.95±0.86 (140.27-143.63)			142.10±1.71 (138.71-145.49)	
6.	Residence	Urban	298 (61.83%)			80 (65.57%)	
		Semi-urban	131 (27.18%)			30 (24.59%)	
		Rural	53 10.99%)			12 (9.84%)	
7.	Intensity of SPT Reactions	Monosensitization	330 (68.46%)			–	
		Disensitization	120 (24.90%)				
		Polysensitzation	32 (6.64%)				
8.	FEV_1_ (Male) (Litre)	Children	Predicted^a^		2.46±0.02 (2.42-2.50)	Predicted^a^	2.63 ±0.06 (2.51-2.75)
			Actual		1.72±0.02 (1.68-1.76)		
		Adolescents	Predicted^a^		3.18±0.02 (3.14-3.22)		
			Actual		2.21±0.03 (2.15-2.27)		
		Young adults	Predicted^a^		3.28±0.02 (3.25-3.31)	Actual	2.39±0.06 (2.27-2.51)
			Actual		2.07±0.02 (2.02-2.12)		
		Old adults	Predicted^a^		2.40±0.03 (2.34-2.46)		
			Actual		1.50±0.03 (1.45-1.55)		
	FEV_1_ (Female) (Litre)	Children	Predicted^b^		2.29±0.01 (2.26-2.32)	Predicted^b^	2.23±0.03 (2.17-2.29)
			Actual		1.54±0.01 (1.51-1.57)		
		Adolescents	Predicted^b^		2.61±0.02 (2.58-2.64)		
			Actual		1.83±0.03 (1.77-1.89)		
		Young adults	Predicted^b^		2.62±0.01 (2.59-2.65)	Actual	2.02±0.03 (1.95-2.09)
			Actual		1.69±0.02 (1.65-1.73)		
		Old adults	Predicted^b^		1.98±0.03 (1.92-2.04)		
			Actual		1.22±0.03 (1.17-1.27)		
9.	FEV1/FVC(% predicted) (Male)	Children	67.51±0.18 (67.16-67.86)			83.04±0.62 (81.81-84.27)	
		Adolescents	59.80±0.93 (57.92-61.68)				
		Young adults	54.98±0.58 (53.82-56.14)				
		Old adults	50.29±1.01 (48.25-52.33)				
	FEV1/FVC(% predicted) (Female)	Children	67.89±0.16 (67.57-68.21)				
		Adolescents	60.11±0.89 (58.30-61.92)				
		Young adults	55.46 ±0.63 (54.21-56.71)				
		Old adults	51.40±0.75 (49.89-52.91)				
10.	PEFR (Male) (Litre/min)	Children	Predicted^c^	403.75±2.13 (399.50-408)		Predicted^c^	428.26±5.69 (416.85-439.67)
			Actual	281.88±2.15 (277.59-286.17)			
		Adolescents	Predicted^c^	475.10±1.73 (471.60-478.60)			
			Actual	330.01±3.89 (322.15-337.87)			
		Young adults	Predicted^c^	483.22±2.07 (479.10-487.34)		Actual	389.15±5.06 (379.01-399.29)
			Actual	315.37±3.17 (309.08-321.66)			
		Old adults	Predicted^c^	414.19±2.52 (409.12-419.26)			
			Actual	279.40±1.82 (275.73-283.07)			
	PEFR (Female) (Litre/min)	Children	Predicted^d^	279.66±1.50 (276.66-282.66)		Predicted^d^	302.53±2.68 (297.19-307.87)
			Actual	188.78±1.74 (185.29-192.27)			
		Adolescents	Predicted^d^	325.84±1.92 (321.95-329.73)			
			Actual	227.85±3.55 (220.66-235.04)			
		Young adults	Predicted^d^	339.83 ±1.32 (337.21-342.45)		Actual	272.51±2.91 (266.71-278.31)
			Actual	219.27±2.18 (214.93-223.61)			
		Old adults	Predicted^d^	287.22±2.46 (282.26-292.18)			
			Actual	176.31±2.76 (170.74-181.88)			

*P value significant.

^a^Predicted FEV1 for male (Litre)= –1.7649+ (–0.0218*age) + (0.0337*height in cm).

^b^Predicted FEV1 for female (Litre)= 0.0381+ (–0.0197*age) + (0.0196*height in cm) (28).

^c^Predicted PEFR for male (Litre/min)= −1.807*age + 3.206*height in cm.

^d^Predicted PEFR for female (Litre/min)= −1.454*age + 2.368*height in cm (23).

### Sensitization of patients against pollens by SPT

**Figure 2:**
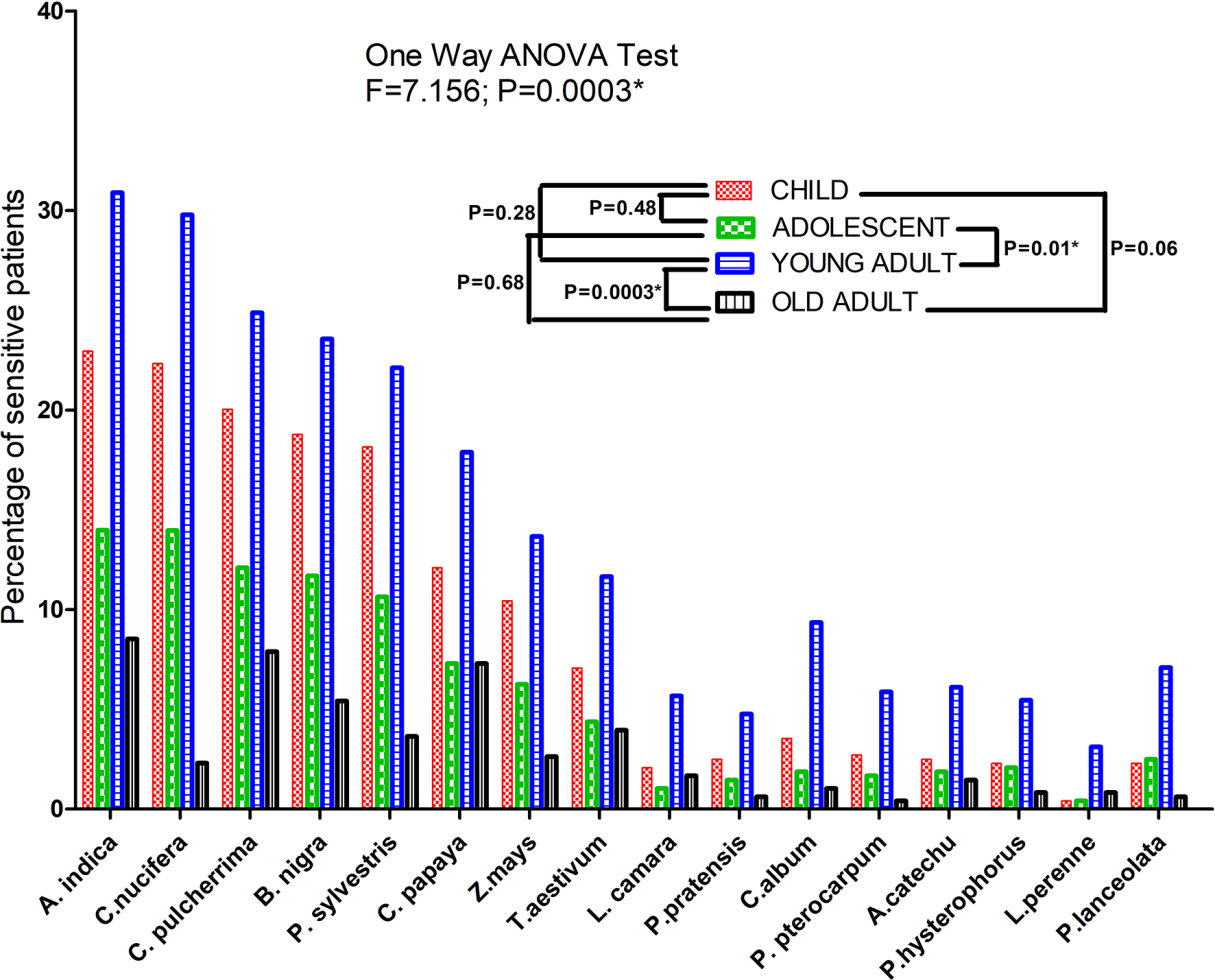
SPT sensitization profile of bronchial asthma patients against different pollen allergen sources. Each bar represents percentage of sensitive patients which is divided according to three age groups. One Way ANOVA revealed a significant difference of SPT sensitivity among different age groups. For pairwise comparison, Post hoc Tukey’s Multiple Comparison test applied which showed significant difference of sensitivity between adolescent and adult. (*P value significant).

The highest prevalence of sensitization was obtained with *Azadirachta indica (87.68%)*, followed by *Cocos nucifera* (83.30%), *Caesalpinia pulcherrima* (74.95%), *Brassica nigra* (72.23%), etc. Sensitivity of patients to different pollen allergens categorized according to age is depicted in **Figure 2**. One-way ANOVA test showed significant difference in sensitivity between the different age groups (F=7.156 and P=0.0003). A pairwise Tukey's Multiple Comparison Test also showed significant difference in number of sensitive patients between adolescent and young adult (P= 0.01) and between young and old adult (P= 0.0003).

### FEV_1_/FVC association with atopy and habitat

**Figure 3:**
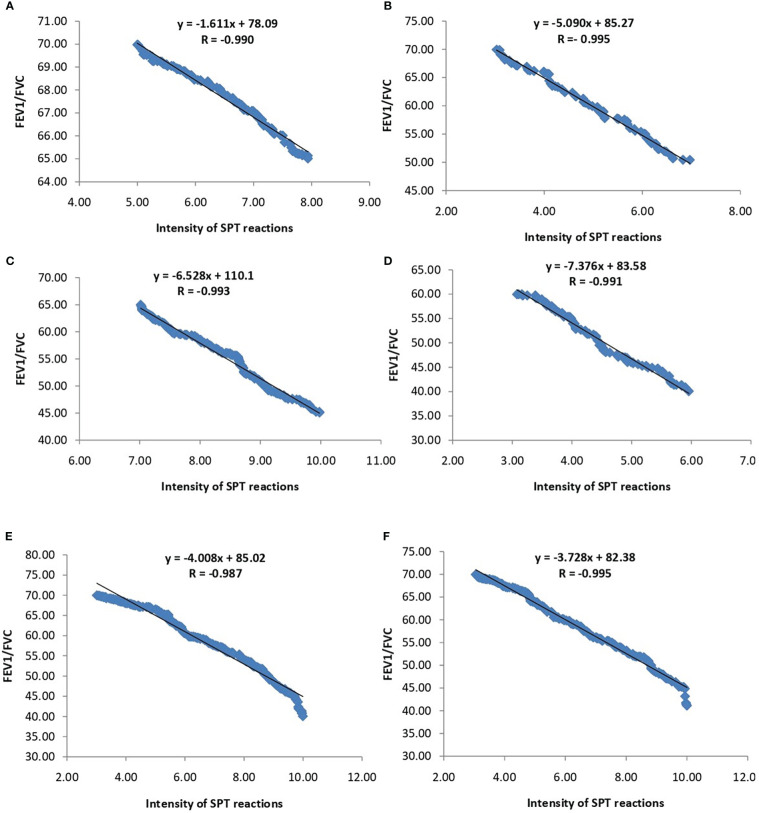
Regression analysis of FEV1/FVC ratio and intensity of SPT reactions (in terms of wheel diameter). **(A)** Children **(B)** Adolescents **(C)** Young adults **(D)** Old adults **(E)** Male **(F)** Female.

**Figure 4:**
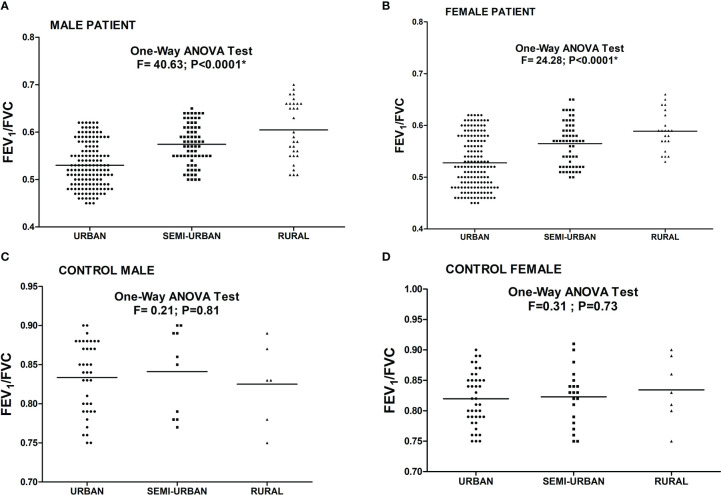
Level of FEV1/FVC ratio in asthmatic patients [**(A)** Male **(B)** Female] and controls [**(C)** Male **(D)** Female] residing in different habitats. One Way ANOVA revealed a significant difference in FEV1/FVC ratio in male and female patients residing in different habitats. (*P value significant).

The association of FEV1/FVC ratio with atopy is depicted in **Figure 3**. Significant negative correlation was found between FEV1/FVC ratio and intensity of SPT reactions which is denoted by wheel diameter in all age groups and sexes (P<0.0001).The association of FEV1/FVC ratio with habitat of both patients and controls is depicted in **Figure 4**. One-way ANOVA test revealed that significant difference existed in FEV1/FVC ratio of both male and female patients residing in different habitats (Urban, semi-urban and rural) (F=40.63 and P<0.0001 and F=24.28 and P<0.0001 respectively); while that in control males and females, it remained non-significant (F=0.21 and P=0.81 and F=0.31 and P=0.73 respectively). Urban patients showed the lowest FEV1/FVC value than others.

### Genotype distribution in patients and controls

The genotype frequencies differ significantly between the study groups (χ2 =7.03; P=0.03) (**Table 3A**). The allele frequencies showed no significant deviation between case and control group (χ2 =2.67; P=0.10).There was no significant deviation from HWE in the control population (χ2 =1.56 at df=1). The frequency of the rs34377097 TT genotype was significantly higher in asthma patients than in controls (OR=5.81, P=0.03). Both recessive and dominant models showed significant difference of rs34377097 TT genotype frequency between the study groups (P=0.04). **Table 3B** shows genotypic frequency distribution of both patients and controls according to age groups (5-12 yrs, 13-19 yrs, 20-40 yrs and 41-70 yrs). The genotype frequencies differed significantly among all the age groups of patients and controls (χ2 =57.02; P<0.0001).The rs34377097 GT and TT genotype bears significant risk of asthma in children of age 5-12 years (OR=2.34, P=0.006 and OR=49.40, P=0.007 respectively). **Table 3C** depicts frequency distribution of both patients and controls according to sex. The genotype frequencies differed significantly between both sexes of patients and controls (χ2 =16.98; P=0.009). The rs34377097 TT genotype showed significant odds ratio in females (OR=12.77, P=0.02). **Table 3D** explains frequency distribution of both patients and controls residing in different habitats (Urban, Semi-urban and rural). The genotype frequencies differed significantly between patients and controls of all habitats (χ2 =29.92; P=0.0009). The rs34377097 TT genotype showed significant risk of asthma in urban and semi-urban dwelling subjects (OR=10.72, P=0.03 and OR=4.89, P=0.02 respectively).

**Table 3:** Genotypic frequency distribution of TBXA2R rs34377097 polymorphism in cases and controls.

Genotype	Model	Patients (n = 482)					Control (n = 122)						Chi Square Value (P value)	Odds Ratio (95% CI)							P value							MAF
Overall distribution																												
**GG**	Additive	230 (47.72%)					76 (62.30%)						7.03 (P=0.03*)	1.00							–							0.20
**GT**		211 (43.78%)					44 (36.07%)							1.58(0.88 to 2.82)							0.12							
**TT**		41 (8.51%)					2 (1.64%)							5.81(1.20 to 28.16)							0.03*							
**GG+GT**	Recessive	441 (91.49%)					120 (98.36%)						4.71 (P=0.03*)	1.00							–							
**TT**		41 (8.56%)					2 (1.64%)							4.85 (1.02 to 23.03)							0.04*							
**GG**	Dominant	230 (47.72%)					76 (62.30%)						3.96 (P=0.04*)	1.00							–							
**GT+TT**		252 (52.28%)					46 (37.70%)							1.77 (1.01 to 3.10)							0.04*							
**G**		671 (69.61%)					196 (80.33%)						2.67 (P=0.10)	1.00							–							
**T**		293 (30.39%)					48 (19.67%)							1.71							0.10							
Distribution according to age																												
		**5-12 yrs**	**13-19 yrs**		**20-40 yrs**	**41-70 yrs**	**5-12 yrs**	**13-19 yrs**		**20-40 yrs**		**41-70 yrs**	57.02 (p<0.0001*)	**5-12 yrs**	**13-19 yrs**		**20-40 yrs**		**41-70 yrs**		**5-12 yrs**	**13-19 yrs**		**20-40 yrs**		**41-70 yrs**		
**GG**	**-**	57 (44.53%)	37 (46.84%)		89 (48.37%)	47 (51.65%)	13 (72.22%)	18 (60.00%)		28 (60.87%)		17 (60.71%)		1.00	1.00		1.00		1.00		–	–		–		–		
**GT**		52 (40.63%)	35 (44.30%)		82 (44.57%)	42 (46.15%)	5 (27.78%)	11 (36.67%)		17 (36.96%)		11 (39.29%)		2.34 (1.28- 4.30)	1.52 (0.85- 2.71)		1.55 (0.87-2.75)		1.38 (0.79- 2.43)		P=0.006*	P=0.16		P=0.14		P=0.26		
**TT**		19 (14.84%)	7 (8.86%)		13 (7.07%)	2 (2.20%)	0	1 (3.33%)		1 (2.17%)		0		49.40 (2.88-845.87)	3.83 (0.98-14.94)		4.45 (0.88-22.39)		5.86 (0.28-124.75)		P=0.007*	P=0.05		P=0.07		P=0.26		
Distribution according to sex																												
	–	Male			Female		Male				Female		16.98 (P=0.009*)	Male				Female			Male				Female			
**GG**		119 (48.37%)			111 (47.03%)		36 (65.45%)				40 (59.70)			1.00				1.00			–				–			
**GT**		109 (44.31%)			102 (43.20%)		18 (32.73%)				26 (38.81%)			1.81 (1.01 -3.24)				1.41 (0.79 - 2.51)			0.05				0.25			
**TT**		18 (7.32%)			23 (9.75%)		1 (1.82%)				1 (1.49%)			4.74 (0.94- 23.83)				12.77 (1.58-103.30)			0.06				0.02*			
Distribution according to Habitat																												
	–	Urban		Semi-urban		Rural	Urban		Semi-urban			Rural	29.92 (P=0.0009*)	Urban		Semi-urban				Rural	Urban		Semi-urban				Rural	
**GG**		139 (46.64%)		59 (45.04%)		32 (60.38%)	50 (62.50%)		18 (60.00%)			8 (66.67%)		1.00		1.00				1.00	–		–				–	
**GT**		135(45.30%)		57 (43.51%)		19 (35.85%)	29(36.25%)		11 (36.67%)			4(33.33%)		1.68 (0.94- 2.99)		1.59 (0.88- 2.84)				1.22 (0.68-2.19)	0.08		0.12				0.51	
**TT**		24 (8.05%)		15 (11.45%)		2 (3.77%)	1 (1.25%)		1 (3.33%)			0		10.72 (1.30- 88.71)		4.89 (1.29-18.56)				10.04 (0.53- 190.38)	0.03*		0.02*				0.12	

Significant associations are shown in asterisk*.

CI, confidence interval; MA, Minor allele frequency in control group.

### Correlation of polymorphic genotypes with FEV1/FVC ratio and atopy

**Figure 5:**
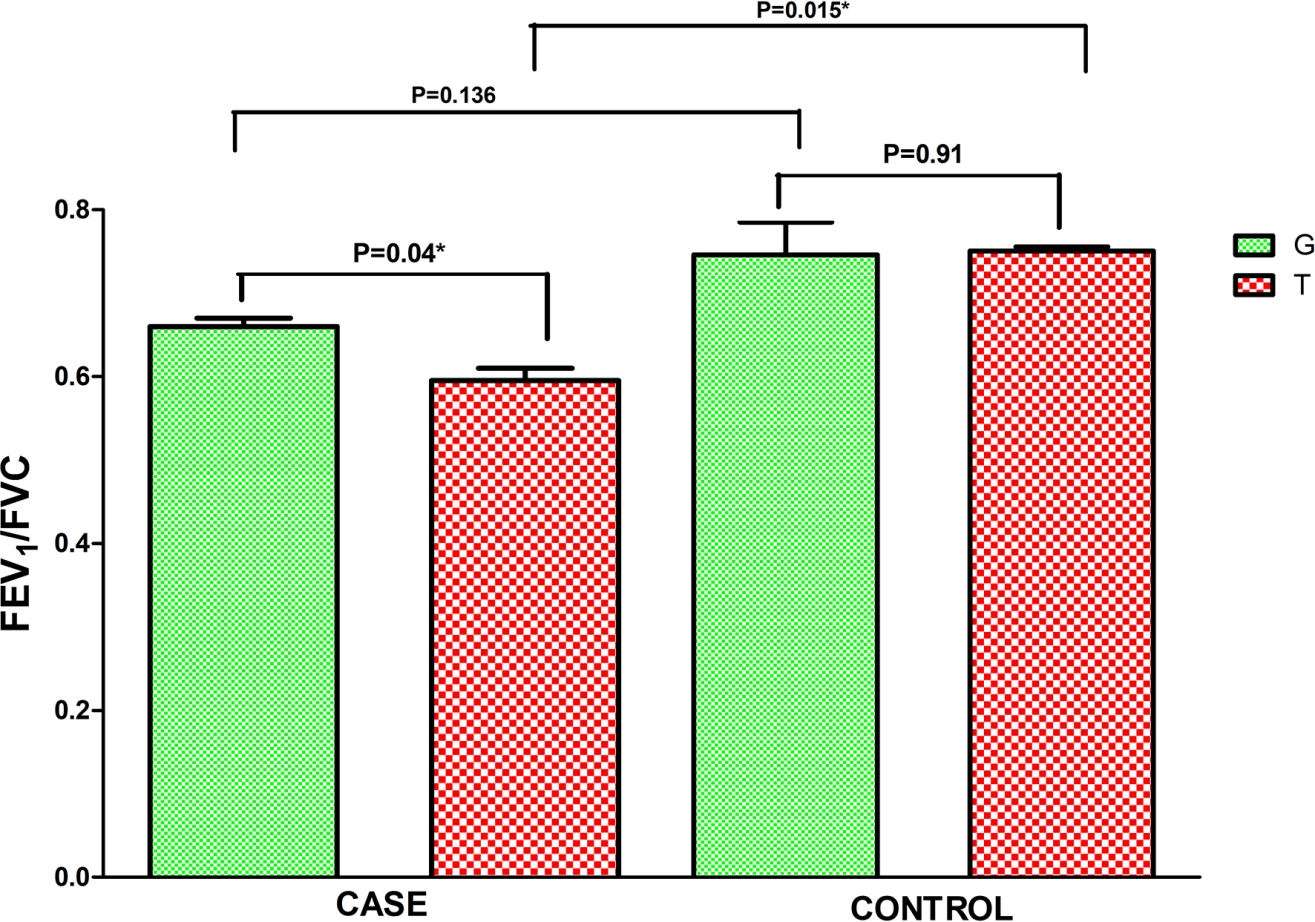
Level of FEV1/FVC ratio in asthmatic patients bearing different alleles of TBXA2R rs34377097 polymorphism. Comparison between cases and controls was performed by Independent t test. (*P value significant).

**Figure 6:**
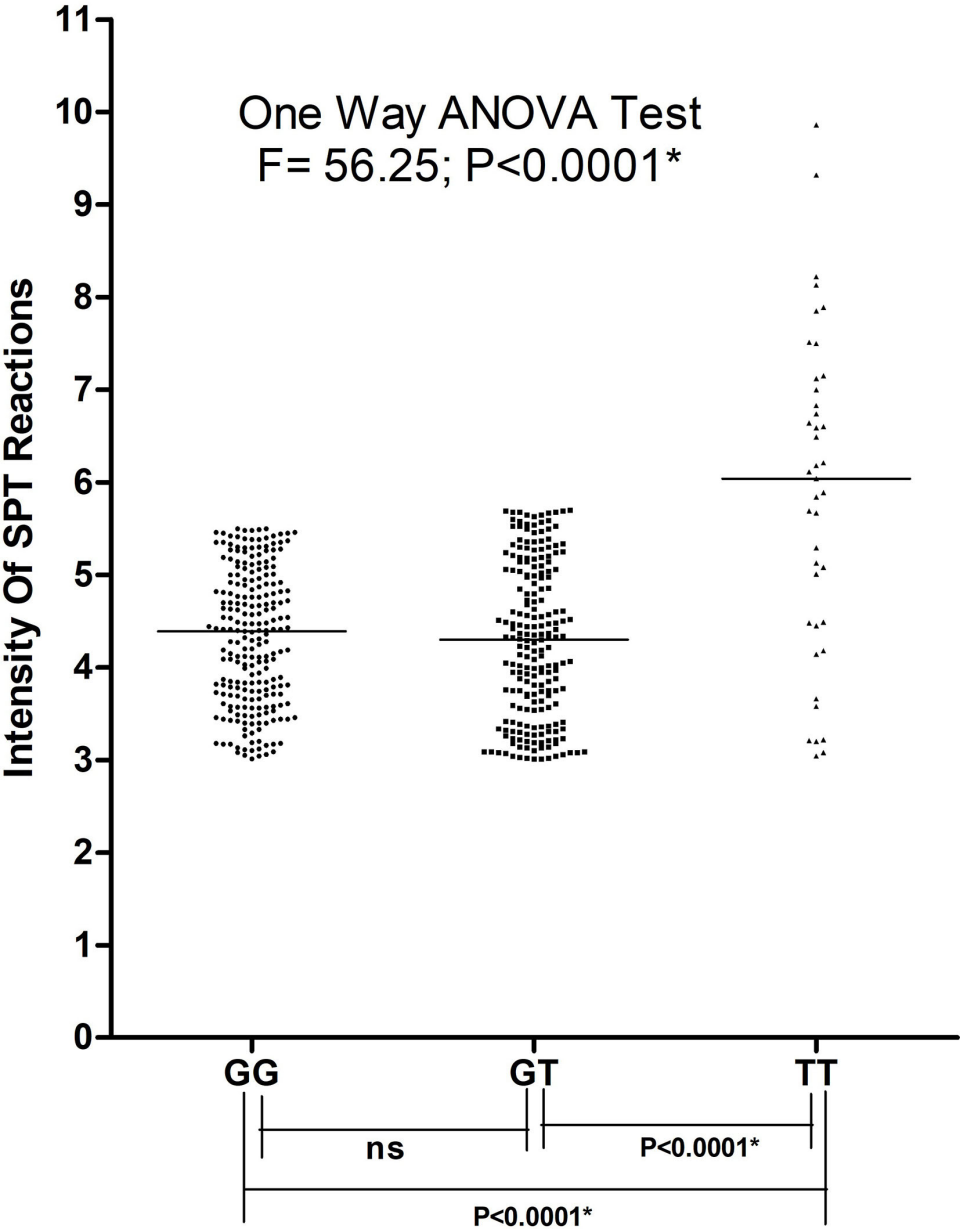
Intensity of SPT reactions in asthmatic patients bearing different genotypes of TBXA2R rs34377097 polymorphism. One-way ANOVA test revealed that individuals bearing TT genotype showed highest intensity of SPT reactions in terms of wheel diameter than GG and GTbearing individuals. (*P value significant).

Asthma patients bearing rs34377097 T allele had significantly lower FEV_1_/FVC ratio (t=8.004, P=0.015) than T allele bearing controls; while that in G allele bearing patient and control individuals remained non-significant (t=2.425, P=0.136) (**Figure 5**). Furthermore, FEV_1_/FVC ratio differ significantly between G and T bearing asthma patients (P=0.04) while no significant difference is observed in controls (P=0.91) (**Figure 5**). One-way ANOVA test revealed that individuals bearing TT genotype showed highest intensity of SPT reactions in terms of wheel diameter than GG and GT bearing individuals (F=56.25 and P<0.0001) (**Figure 6**).

### Mechanistic and structural analysis of asthmatic response induced by rs34377097

**Figure 7:**
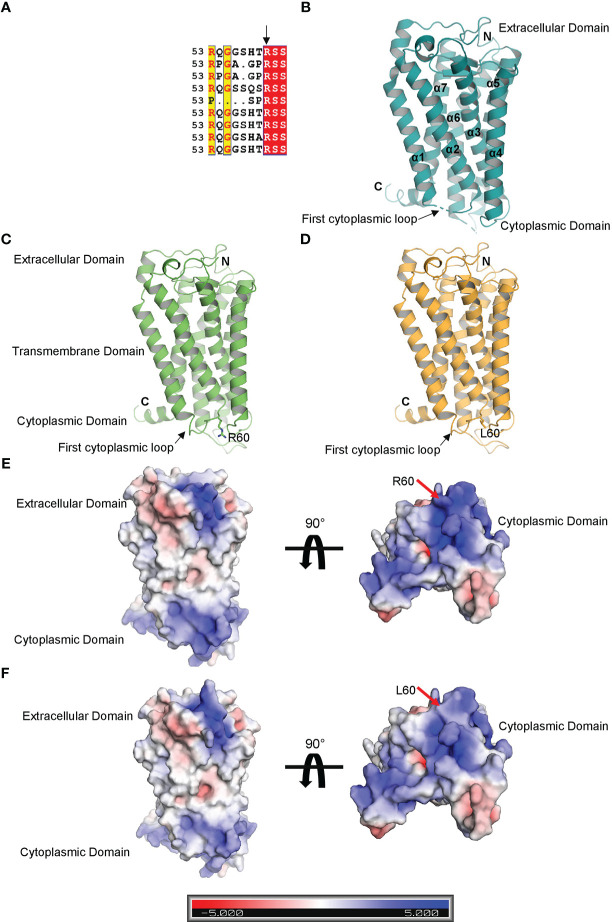
Structural analysis of TBXA2R and TBXA2R R60L. **(A)** Multiple sequence alignment of TBXA2R mammalian orthologs. **(B)** Cartoon representation of the crystal structure of human TBXA2R(PDB: 6IIU). Electron density deficient loops are shown with dotted lines. **(C)** Cartoon representation of 3-D Structural model of human TBXA2R depicted in smudge. R60 residue is depicted in stick. **(D)** Homology model of TBXA2R R60L is depicted in bright orange. L60 residue is shown in stick.Electrostatic surface potential of Wild type TBXA2R **(E)** and TBXA2R R60L **(F)** are coloured according to the bar underneath.The scale ranges from −5 kT/e (red) to +5 kT/e (blue).

Multiple sequence alignment of TBXA2R gene among the orthologs in mammals (*Mus musculus, Rattus norvegicus, Bos taurus, Mesocricetus auratus, Macaca mulatta, Pan troglodytes, Felis catus, Chlorocebus aethiops*) denoted that the R60 amino acid is conserved across the species (**Figure 7A**). The crystal structure of human TBXA2R (PDB: 6IIU) revealed that the structures share a canonical seven-transmembrane helical bundle similar to other known GPCR structures (**Figure 7B**) (29). The N-terminal extracellular domain functions as a receiver domain, whereas the C-terminal cytoplasmic domain functions as a signal transmitter domain. The Guanine nucleotide-binding Gq protein binds at the C-terminal cytoplasmic domain of TBXA2R and relays the signal. In the crystal structure of human TBXA2R, a number of important cytoplasmic loops, including the first cytoplasmic loop that contains the R60 residue, were lacking their electron densities (shown by dotted lines in **Figure 7C**). In order to model the cytoplasmic loops, we have prepared a structural model of TBXA2R using the artificial intelligence (AI) based tool *AlphaFold2 (* 30). Superimposition of the model and the crystal structure (PDB: 6IIU) yielded root-mean-square deviations of 0.049Å. In order to understand the effect of R60L, we have prepared a homology model of the mutated TBXA2R protein. **Figure 7D** shows presence of L60 amino acid at the first cytoplasmic loop. We have generated the electrostatic potential surface of wild type TBXA2R (**Figure 7E**) and TBXA2R R60L protein (**Figure 7F**). It is interesting to note that the TBXA2R protein in its wild type exhibits strong positive charge potential (depicted in blue) on its cytoplasmic surface (**Figure 7E**). Hence, it can be assumed that proteins that interact with the cytoplasmic domain of TBXA2R must possess negative charge potentiality. Further, the cytoplasmic domain of TBXA2R R60L displays much less positive charge potentiality (location is marked with red arrow) as compared to the wild type protein (**Figure 7F**). This is because Arg is a positively charged amino acid whereas Leu is a hydrophobic amino acid. The interaction between TBXA2R and the Gq protein may be inhibited if the positive charge potential at the cytoplasmic domain of TBXA2R is reduced.

## Discussion

Asthma is a heterogeneous and chronic airways disease and estimated to affect an average of 4.5% individuals throughout the globe, with substantial variation in different countries (31). Bronchial asthma is influenced by both environmental and genetic components (32). There is a substantial curiosity in upgrading of our understanding regarding biological mechanism of bronchial asthma by expanding genomic approaches. Current investigation is based on the association of TBXA2R rs34377097 polymorphism in an Indian population.

Earlier reports indicated that TBXA2 was a negative regulator of LTC4 synthase, which produces leukotriene (33). Evidence suggested that in absence of TBXA2, LTC4 synthase activity is enhanced through its receptor (TBXA2R), resulting in increased leukotriene biosynthesis and asthma symptoms (34). These results clearly signify that TBXA2R polymorphism might play an important role in bronchial asthma pathogenesis. TBXA2 is synthesized from arachidonic acid by the enzyme, cyclooxygenase (COX) (35). It causes constriction of bronchial smooth muscles in asthma (36–38). Pollen grains are important aeroallergens therefore it is essential to havethe knowledge of locally prevalent pollen allergens for better diagnosis and therapy of pollen induced asthma (11, 39). Allergenic potentials of common pollens are investigated throughout India viz. *Cynodon* in northern region, *Fusarium solani* and *Curvularia* from southern region, *Azaridacta* in central region, *Cocos* and *Phoenix* from eastern region (40). In our study, more than 87% of patients were sensitive to *Azadirachta indica*, one of the major pollens found in West Bengal. This study also reported *Cocos nucifera, Caesalpinia pulcherrima and Brassica nigra*as top sensitizer as depicted in **Figure 2** which is in accordance to reports from Dey et al. (2019) (14). Podder et al. 2009, 2010 (41, 42) reported that the occurrence of allergic symptoms to various inhalants is similar for both sexes. We also found significant difference in SPT sensitivity between adolescent & young adult and between young & old adult. Dey et al. (2019) (14) also reported similar observations among atopic population of West Bengal, India. This difference in sensitivity may be due to the increased exposure to various pollen allergens in the residential/occupational area encountered by young adults compared to adolescents and old adults. **Figure 3** showed significant negative correlation of FEV1/FVC ratio and intensity of SPT reactions (in terms of wheel diameter) in all age groups and in both sexes which clearly indicated a role of atopy in lung function impairment. Various studies were conducted previously showing association of allergen sensitization and asthma severity in terms of spirometric parameters (43, 44). Jaen et al. 2002 (45) reported that asthmatics sensitized to various allergens including pollens have significantly lower FEV1/FVC ratio which is in accordance with our result. Allergens are thought to initiate acute episodes of asthma through inflammatory processes and bronchial hyper-responsiveness that might result into airway obstruction (46). **Figure 4** depicted existence of significant difference in FEV1/FVC ratio of both male and female patients residing in different habitats (Urban, semi-urban and rural) while that in control subjects remained non-significant. Some previous studies reported that asthma patients residing in urban and semi urban habitat with high indoor and atmospheric pollution show reduced FEV1/FVC ratio than patients from rural counterparts (47, 48) which supported our observation.

**Table 2** showed that 56.43% of our study population inherited asthma from their family (either maternal or paternal or both), which supports the role of genetic predisposition in asthma. Literature suggested that TBXA2R pathway play a significant role in asthma (21, 33). In our study, we found significant association of rs34377097 polymorphic TT genotype in the exon-2 region of the TBXA2R gene with elevated pollen induced asthma response such as acute broncho and tracheal constriction in West Bengal population, India. **Table 3A** clearly demonstrated that TBXA2R rs34377097 TT genotype was significantly associated with pollen induced asthma (OR=5.81, P=0.03). **Table 3B** depicted that children of age 5-12 years bearing GT and TT genotype have higher risk of asthma than adolescent, young adult and old adult. Prashanth et al. 2022 (49) reported mean urinary Leukotriene level in asthmatic children as 378.47 Pg/Mg and Green et al. 2004 (50) observed mean urinary Leukotriene level in asthmatic adult as 111.70 Pg/Mg which may be a probable cause of higher asthma risk in children. Besides this, Kim 2004 (51) reported that children spend more time outdoors than adults, mostly in summer during late afternoon. This long-term exposure to various outdoor air pollutants significantly contributes in developing asthma since their immune system and lungs are still immature. **Table 3C** showed that females bearing TT genotype have significant higher risk of asthma. Pignataro et al. 2017 (52) reported that females have higher risk of developing asthma than males which is in accordance to our result. This difference in asthma risk between female and male may be due to expression of female sex hormone receptors on mast cells (53). In **Table 3D**, we observed that urban and semi-urban dwelling patients bearing TT genotype have higher risk of asthma. Priftis et al. 2009 (54) reported that children living in urban areas have significantly higher risk of asthma than their rural counterparts which confirmed our result. This may be due to increased exposure to indoor and outdoor air pollutants in urban and semi-urban habitat. Emissions of harmful gases from motor traffic contribute significantly to the development of asthma in urban and semi-urban dwelling patients (55).

As per our knowledge is concerned, our study which is based on both structural and functional aspects, is the first one to report the association of TBXA2R rs34377097 polymorphism in an Indian population. It is also the first extensive in silico analysis that perform both polymorphism study and modeling approach to assess TBXA2R rs34377097 polymorphism clinically. Another study conducted in Japanese population reported lack of association of rs34377097 polymorphism with bronchial asthma (21). The present study also depicted significant associations of several clinical parameters like FEV_1_, FEV_1_/FVC ratio and PEFR with pollen induced asthma both in male and female patients in all age groups. Furthermore, in patients the risk allele T was found to be clinically correlated with FEV_1_/FVC ratio (P=0.015). **Figure 6** demonstrated that TBXA2R rs34377097TT genotype bearing individuals showed highest intensity of SPT reactions in terms of wheel diameter than GG and GT bearing individuals which indicate association of atopic status with asthma risk.

**Figure 8:**
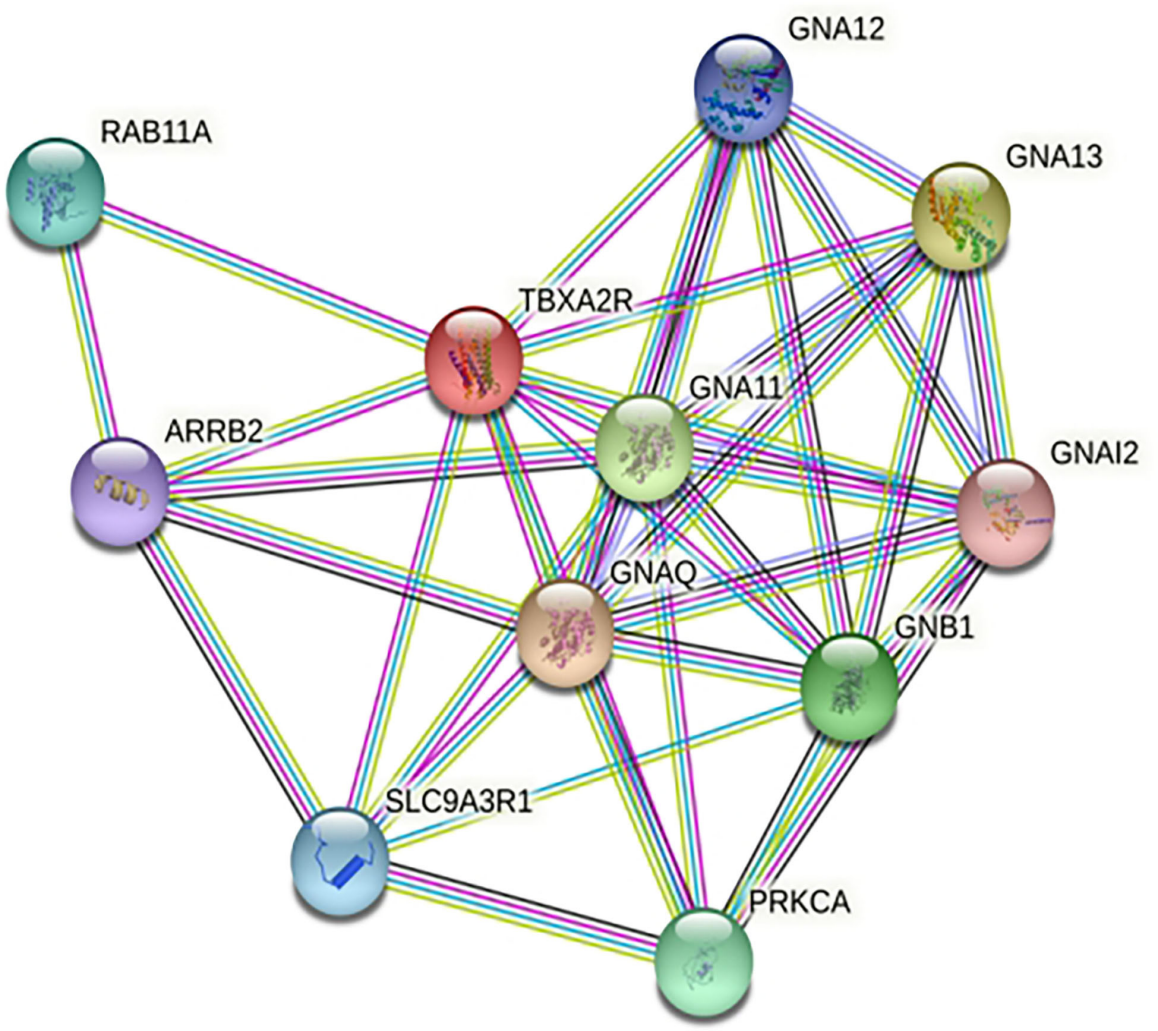
Protein- protein interaction network of TBXA2R.

**Figure 9:**
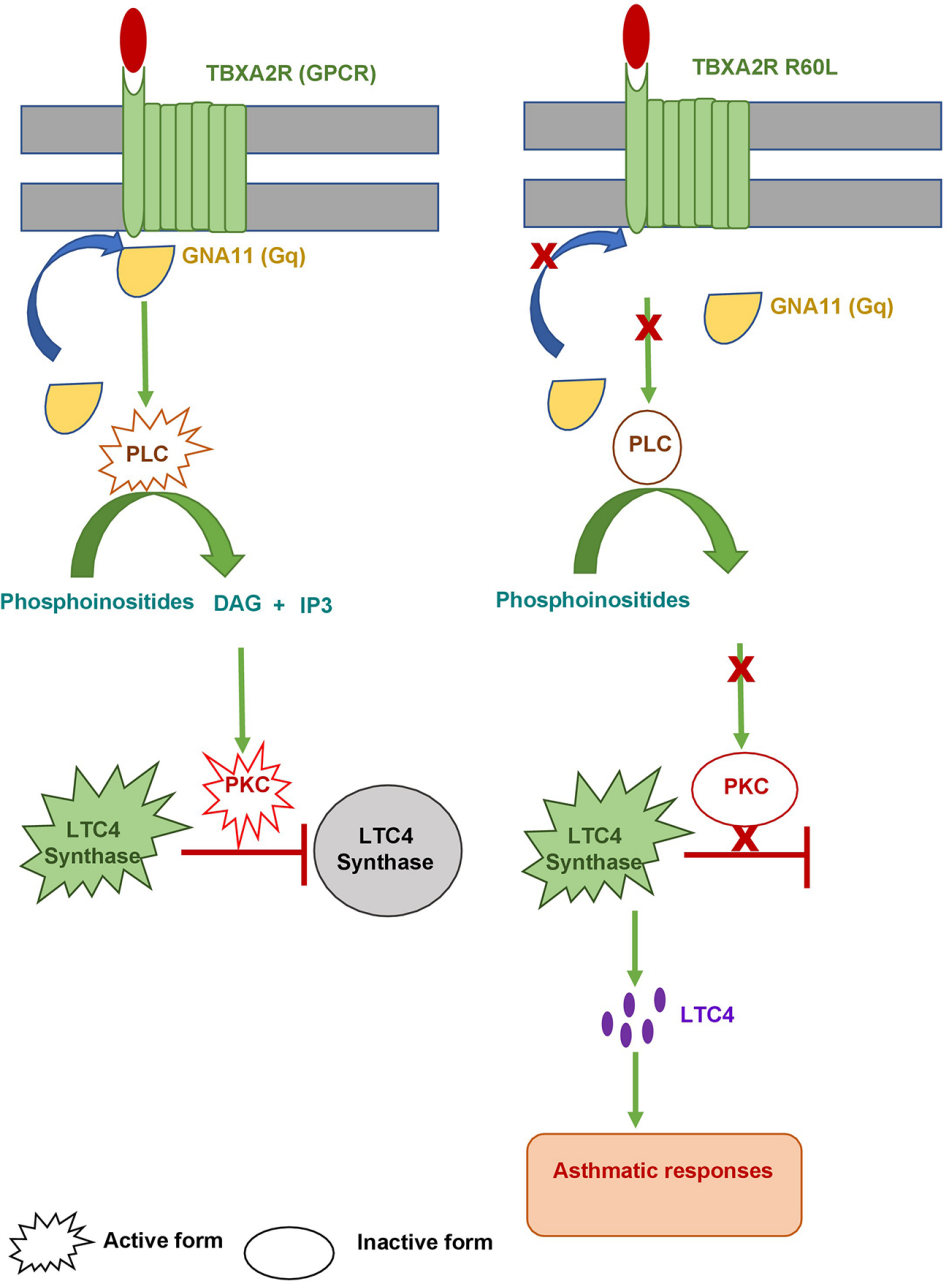
Model for asthmatic regulation *via*. TBXA2R. The left panel shows wild type TBXA2R prevents asthmatic responses by converting active LTC4 Synthase into an inactive one. The right panel shows how a mutation (R60L) in TBXA2R causes an asthmatic response.

STRING analysis showed that TBXA2R are involved in interaction with different known and predicted Gq proteins (guanine nucleotide-binding G protein, subunits alpha, group q) (**Figure 8**). Out of all Gq proteins, GNA11 acts as an activator of phospholipase C (PLC). PLC hydrolyses phosphoinositides into the two stimulatory second messengers - inositol 1,4,5-triphosphate (IP3) and diacylglycerol (56). IP3enhances cytoplasmic free calcium level and diacylglycerol (DAG) activates protein kinase C (PKC). Activated protein kinase C either directly phosphorylates LTC_4_ synthase enzyme and inactivates it or this regulation may involve another regulatory protein which is yet to be discovered. Inactivation of LTC_4_ synthase leads to reduction of Leukotriene C_4_ (LTC_4_) biosynthesis in platelets. However, in the case of risk allele rs34377097 T-bearing individuals, the non-synonymous mutation (R60L) in TBXA2R protein inhibits the interaction between GNA11 and TBXA2R due to the change in the positive charge potentiality at the cytoplasmic domain. This is in line with Hirata et al. 1996 (57), who demonstrated that the R60L mutation significantly reduces PLC activity. That leads to simultaneous inactivation of PKC which ultimately results in LTC_4_ synthase enzyme activation. Activation of this enzyme leads to LTA_4_ to LTC_4_ conversion which results in acute asthmatic response including broncho-constriction, tracheolar constriction and increased mucus secretion as shown in **Figure 9**.

## Conclusion

The present study provided several lines of evidence to support that TBXA2R rs34377097 polymorphism act as a potent risk factor for asthma in West Bengal population, India. More studies are warranted in diverse ethnic groups to validate the above findings. The present in-silico study will be useful for the advancement of gene based therapy for better management and treatment of asthma in near future.

## Data availability statement

The datasets presented in this study can be found in online repositories. The names of the repository/repositories and accession number(s) can be found below: SCV003761534 (ClinVar).

## Ethics statement

The studies involving human participants were reviewed and approved by Clinical Research Ethics Committee, Allergy and Asthma Research Center, West Bengal, India (CREC-AARC Ref: 62/21). Written informed consent to participate in this study was provided by the participants’ legal guardian/next of kin.

## Author contributions

Collection of samples was done by IG, SS and NS. IG and AL contributed to data compilation and wrote first draft of the manuscript. PB did the structural modeling. SM diagnosed the disease and provided the samples. Statistical analysis was done by IS, HB and AL. SP conceived the study design, supervised the work, critically revised the manuscript and made the final draft. All authors contributed to the article and approved the submitted version.
